# Long-term comorbidity associated with childhood periodic fever, aphthous stomatitis, pharyngitis, and adenitis (PFAPA) syndrome: a case–control study

**DOI:** 10.1007/s00431-026-07156-2

**Published:** 2026-06-12

**Authors:** Henna Uosukainen, Sallamaaria Kettunen, Terhi Ruuska-Loewald, Sauli Palmu, Sakari Linnola, Marjo Renko, Ulla Lantto

**Affiliations:** 1 Kuopio Pediatric Research Unit (KuPRU), University of Eastern Finland and Kuopio University Hospital, Kuopio, Finland; 2 Department of Pediatrics, Päijät-Häme Central Hospital, Lahti, Finland; 3 Clinical Medicine Research Unit and Biocenter Oulu, University of Oulu, Oulu, Finland; 4 Pediatrics and Adolescent Medicine, Oulu University Hospital, Oulu, Finland; 5 Otorhinolaryngology and Head and Neck Surgery, Oulu University Hospital, Oulu, Finland; 6 Center for Child, Adolescent and Maternal Health Research, Faculty of Medicine and Health Technology, Tampere University, Tampere, Finland; 7 Department of Pediatrics, Tampere University Hospital, Wellbeing Services of Pirkanmaa, Tampere, Finland

**Keywords:** Comorbidity, Infection, Otitis, PFAPA, Periodic fever, Risk factors

## Abstract

The aim of this study was to evaluate the long-term morbidity of patients with PFAPA compared with controls selected from the general population. We identified 244 patients treated for PFAPA at Tampere, Oulu, and Kuopio University hospitals between 2008 and 2018. In May 2023, and January 2024, these patients were invited to complete a detailed questionnaire regarding their past and current health and lifestyle. A total of 2433 controls, matched for age, sex, and birthplace, were randomly selected from the Population Register Center of Finland and completed the same survey. We compared the occurrence of acute and chronic illnesses and childhood environmental exposures between PFAPA patients and controls 5–16 years after the initial PFAPA diagnosis. Responses were obtained from 98 PFAPA patients (40.1%) and 490 controls (20.1%). Atopy was the only chronic condition significantly more common among PFAPA patients than controls (14.3% vs 7.9%; OR 2.0, 95% CI 1.0–3.9). PFAPA patients more frequently reported recurrent otitis media (36.2% vs 20.9%; OR 2.3, 95% CI 1.4–3.8), tympanostomy (24.5% vs 15%; OR 1.9, 95% CI 1.1–3.2), bronchitis (4.2% vs 1.2%; OR 4.0, 95% CI 1.1–14.9) and hospitalization due to infections (30.2% vs 17.9%; OR 1.9, 95% CI 1.2–3.2). Fungal infections were also more common among PFAPA patients (8.5% vs 3.7%; OR 2.4, 95% CI 1.0–5.9). No differences were found in other chronic conditions or environmental exposures.

*Conclusion*: Children with PFAPA did not exhibit greater long-term morbidity than controls, except for a higher prevalence of atopy. Respiratory infections, recurrent otitis media, and fungal infections were more frequent among cases than controls. These findings may reflect greater healthcare use rather than true susceptibility to infections. Environmental risk factors were similar between the groups.

**Table:** 

**What is Known:** • *Current evidence does not suggest an association between PFAPA and other chronic conditions.* • *Prior evidence on infection susceptibility in PFAPA has been inconsistent.*	
**What is New:** • *Children with a history of PFAPA show no increased long-term morbidity except for atopy.* • *PFAPA patients have more childhood AOM, bronchitis, and fungal infections than controls from the general population. Increased rates of diagnosed infections and hospitalizations in PFAPA patients may be influenced by healthcare‑seeking behavior and diagnostic practices, rather than reflecting true infection susceptibility.*

**What is Known:**

• *Current evidence does not suggest an association between PFAPA and other chronic conditions.*

• *Prior evidence on infection susceptibility in PFAPA has been inconsistent.*

**What is New:**

• *Children with a history of PFAPA show no increased long-term morbidity except for atopy.*

• *PFAPA patients have more childhood AOM, bronchitis, and fungal infections than controls from the general population. Increased rates of diagnosed infections and hospitalizations in PFAPA patients may be influenced by healthcare‑seeking behavior and diagnostic practices, rather than reflecting true infection susceptibility.*

## Introduction

Periodic fever, aphthous stomatitis, pharyngitis, and cervical adenitis (PFAPA) is an autoinflammatory syndrome predominantly affecting children under five. First described by Marshall et al. in 1987 [1], PFAPA is the most common cause of recurrent fever in children, with a cumulative incidence of 2.3 cases per 10,000 children younger than 5 years [2].

In PFAPA, fever episodes come periodically, circa once a month lasting for 3–6 days. Notably, patients experience periods of good health between episodes. Due to lack of specific PFAPA tests, diagnosis is based on clinical evaluation and exclusion of other conditions causing recurrent fever [3, 4]. Tonsillectomy has been found to be an effective treatment; after surgery, fever episodes stop completely in almost all patients [5, 6]. Without any intervention, the syndrome tends to resolve spontaneously. However, this can take several years [2, 4, 7].

The etiology of the disease has long been investigated. The autoinflammatory disease label is supported by activation of the immune system during fevers and corticosteroid effectiveness in extinguishing febrile episodes [8, 9]. Inflammasomes, involved in various autoinflammatory responses, may be activated by microbes or other environmental stimuli [10, 11].

Several studies have reported equal or even lower susceptibility to infections between patients with PFAPA and controls [4, 12, 13]. In contrast, in our previous study, we reported significantly greater quantity of respiratory infections, otitis media, and oral thrush in PFAPA cases, along with increased hospital visits and antibiotic use [2, 14]. Patients with PFAPA have been reported of having similar rates of autoimmune and chronic diseases as well as similar growth rates compared to controls [14, 15]. Regarding environmental and lifestyle factors, maternal smoking and lack of breastfeeding have been observed to be more common in patients with PFAPA. No additional environmental factors have been associated with the syndrome, except for the aquarium at home [16].

To further elucidate the overall long-term morbidity and environmental risk factors associated with PFAPA syndrome, we analyzed these issues in a new case–control study.

## Materials and methods

### PFAPA patients

**Fig. 1:**
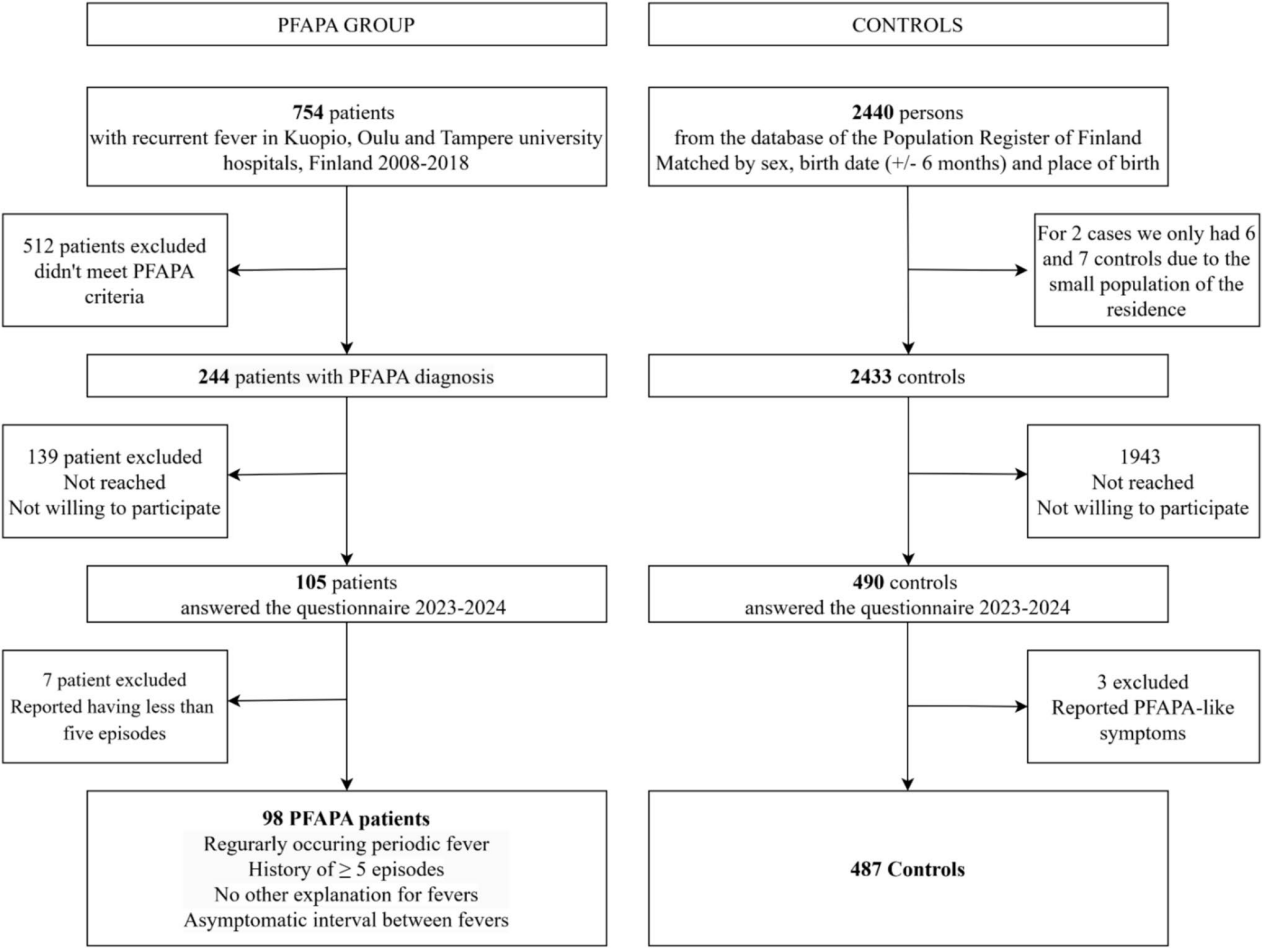
Flowchart illustrating the identification of PFAPA patients and matched controls, including response rates and exclusion criteria

The cohort was assembled retrospectively based on medical record review of PFAPA patients from three Finnish hospital regions from 2008 to 2018. Diagnostic and clinical information had been recorded contemporaneously during routine clinical care and was subsequently extracted retrospectively for the purposes of this study. As the syndrome does not have a specific ICD-code, we collected data on all patients under 10 years of age attending Kuopio, Oulu, or Tampere University hospitals for recurrent fever. Patients were identified in the hospital information system using visit records from pediatric and otorhinolaryngology outpatient clinics, applying the following ICD‑10 codes: R50.9 (fever, unspecified), A68.9 (relapsing fever, unspecified), and J35.0 (chronic tonsillitis). We reviewed their medical records to determine whether they met the diagnostic criteria for PFAPA [17]. Most patients had received a clinical diagnosis of PFAPA during routine care; 95.1% had a recorded PFAPA diagnosis, while in the remaining cases, the diagnosis was confirmed retrospectively based on documented clinical features. At the time of patient identification, PFAPA diagnoses were made according to prevailing clinical practice, which largely reflected the original Marshall description. Due to the retrospective nature of the study and incomplete documentation of some clinical features, formal retrospective application of the full Marshall or Eurofever/PRINTO criteria was not feasible for all patients. Eligible cases had experienced at least five regularly occurring fever episodes, were asymptomatic between the episodes, and had no alternative explanatory diagnosis in the medical records. A total of 244 patients with PFAPA syndrome were identified. Follow‑up was conducted retrospectively using a cross‑sectional survey administered during a fixed calendar period (May 2023–May 2024). We contacted these patients by mail and provided an information letter containing a link and QR code to an electronic survey. All follow‑up outcomes were collected via a self‑reported questionnaire completed by patients or their caregivers. Outcome data were not systematically validated against medical records or registers. Patients were contacted regardless of the year of diagnosis, resulting in variable follow‑up durations. Of the 105 responses received (43%), seven were excluded because fewer than five fever episodes were reported (Fig. 1).

### Control subjects

Controls were randomly selected from the Population Register Center of Finland and matched to PFAPA patients by sex, birth date (± six months), and place of residence at birth. We approached 10 potential controls per case by mailed letter containing the same link as cases, which they answered anonymously. We contacted 2433 potential controls by mail and received 490 responses (20%). We excluded three controls because of PFAPA-like symptoms in their history (Fig. 1).

### Questionnaires

We collected answers from both groups between May 2023 and May 2024. The questionnaire consisted of 58 questions, 14 of which were PFAPA‑specific questions addressing the history of fever episodes, classic symptoms of PFAPA and family history of recurrent fevers. The questionnaire included detailed questions about child’s history of common childhood infections, physician-diagnosed diseases, allergies, surgical procedures, and yeast or fungal infections. In addition, we asked about the duration of breastfeeding, daycare attendance, household smoking habits, use of vitamins and natural products, and exposure to domestic or farm animals during childhood. We also collected information about the number of siblings and parents' education.

The study was conducted in accordance with the ethical standards of the regional research ethics committee and with the 1964 Declaration of Helsinki and its later amendments. The research protocol was approved by the Regional Medical Research Ethics Committee of the Wellbeing Services County of North Ostrobothnia. Responding to the electronic questionnaire was considered as a consent to participate in the study.

### Statistical analysis

For continuous variables, we calculated means and standard deviations or medians and ranges, depending on the distribution within each group. Differences between groups were assessed using either the student *t* test or the Mann–Whitney *U* test, depending on the distribution. We compared the proportions between PFAPA patients and controls and calculated their differences with their 95% confidence intervals (CI). We interpreted the statistical significance for differences in proportions using CIs and *χ*^2^ tests. To analyze if any of the factors with significant difference in the univariate analyses were independently associated with PFAPA syndrome, we conducted a multivariate logistic regression analysis. The model was adjusted for family socioeconomic status (derived from fathers’ education), sex, and participants’ age at the time of the questionnaire. Although controls were matched to PFAPA patients at sampling based on age, sex, and place of birth, analyses were conducted treating the groups as independent samples. This approach was chosen because differential response rates prevented preservation of matched pairs. Age and sex were therefore included as covariates in regression models. Methods for matched data, such as conditional logistic regression, were not applied as pairwise matching could not be maintained in the final analytical sample. We estimated odds ratios (ORs) with corresponding CIs for each factor. Adjusted odds ratios (aORs), along with their CIs and *p*-values, are reported. We considered values of *p* < 0.05 statistically significant. We performed all analyses using IBM SPSS Statistics for Windows, version 29.0 (IBM Corp., Armonk, NY, USA).

## Results

The final data consisted of 98 patients with PFAPA, of whom 45 (45.9%) were male. The mean age at PFAPA diagnosis was 4.2 years (SD 2.6) and at the time of the questionnaire 14.0 years (3.4 SD). The median follow‑up time was 9.4 years (range 5.0–15.7 years). Tonsillectomy had been performed in 70 of 98 patients (71.4%). At follow‑up, five patients (15.2%) reported continued recurrent febrile episodes, while the majority reported resolution of fever symptoms. Among 96 respondents, 24 (24.5%) reported suspected PFAPA in a first‑degree relative. The control group consisted of 487 children matched for age, sex and place of residence at birth; 239 (49.1%) were male and the mean age at the time of questionnaire was 13.9 years (Table 1). The questionnaires were completed by parents or with parental assistance in 501 of 585 cases (85.6%).

**Table 1:** Characteristics of children with a history of PFAPA and matched population-derived controls (matched for age at data collection, sex and family social class based on the father’s education). Sample size varies in the PFAPA group (*n* = 96–98)

Characteristics	PFAPA group, *n* = 98	Control group, *n* = 487
Age at the time of PFAPA diagnosis, mean (SD)	4.21 (2.55)	NA
Age, at the time of the questionnaire, years, mean (SD), median (range)	14.0 (3.4), 14.1 (7.2–23.8)	13.9 (3.6), 13.4 (6.9–23.8)
Follow-up time, years, median (range)	9.4 (5.0–15.7)	NA
Sex, male, *n* (%)	45 (45.9)	239 (49.1)
Number of fever episodes at the time of PFAPA diagnosis, *n* (%)		
- 5–10 episodes	54 (55.1)	NA
- > 10 episodes	42 (42.9)	NA
Symptoms at the time of PFAPA diagnosis,* n* (%)		
- Aphthous stomatitis at the time of fever	14 (14.3)	NA
- Pharyngitis at the time of fever	54 (55.1)	NA
- Adenitis at the time of fever	55 (56.1)	NA
- Fever as the only symptom of PFAPA	30 (30.6)	NA
PFAPA was treated with tonsillectomy, *n* (%)	70 (71.4)	NA
- Fever episodes ceased after tonsillectomy,* n* (%)	63 (94.0)	NA
PFAPA cases in first-degree relatives, *n* (%)	24 (24.5)	NA
Infection just before the onset of fevers, *n* (%)	16 (16.3)	NA
Treatment with antibiotics at least once, *n* (%)	49 (50.0)	NA
Father’s education, *n* (%)		*n* = 485
- Primary school	2 (2.0)	16 (3.3)
- High school or equivalent	54 (55.1)	205 (42.1)
- University of Applied Sciences	29 (29.6)	154 (31.6)
- University	13 (13.3)	110 (22.6)
Mother’s education, *n* (%)		
- Primary school	0	4 (0.8)
- High school or equivalent	38 (38.8)	141 (29.0)
- University of Applied Sciences	34 (34.7)	185 (38.0)
- University	26 (26.5)	155 (31.8)

### Long- term morbidity

Regarding chronic illnesses, atopy was reported by 14 of 98 PFAPA cases and in 38 of 482 controls (14.3% vs 7.9%; OR 2.0, 95% CI 1.0–3.9). Children with atopy had more fungal infections in their history as compared to non-atopic children (12% vs 3.8%; OR 3.6, 95% CI 1.3–9.6, data not shown). No other chronic diseases or allergies differed significantly between PFAPA patients and controls (Table 2). Exploratory analyses comparing patients who had undergone tonsillectomy with those whose symptoms resolved spontaneously did not reveal differences in overall morbidity or identified risk factors; however, atopy, food allergies, and history of tympanostomy were more frequently reported among patients who had undergone tonsillectomy.

**Table 2:** Comparing 98 PFAPA patients and 487 controls regarding long-term diseases and operative treatments. Odds ratios are adjusted for age at data collection, sex, and social class of the family based on the father’s education (aOR). *N* may vary in cases (93–98) and in controls (473–487)

	PFAPA group *N* (%), *n* = 98	Controls *N* (%), *n* = 487	*P* value	aOR (95% CI)
Diseases				
Asthma	5 (5.1)	34 (7.1)	0.482	0.653 (0.245–1.742)
Atopy	14 (14.3)	38 (7.9)	0.043	1.994 (1.029–3.865)
IBD	0	3 (0.6)	0.434	–*
Diabetes with insulin therapy	0	1 (0.2)	0.652	–*
Migraine	7 (7.1)	23 (4.8)	0.334	1.472 (0.605–3.581)
Epilepsy	2 (2.0)	0	0.002	–*
Juvenile arthritis	1 (1.0)	1 (0.2)	0.211	4.047 (0.248–66.140)
Other disease diagnosed by a doctor^a^	28 (28.6)	100 (20.7)	0.089	1.542 (0.934–2.546)
Allergies	26 (27.1)	110 (22.8)	0.369	1.348 (0.811–2.239)
Pollen	19 (19.8)	84 (17.4)	0.580	1.269 (0.721–2.235)
Animal	5 (5.2)	37 (7.7)	0.395	0.675 (0.257–1.774)
Food	14 (14.6)	50 (10.4)	0.230	1.553 (0.816–2.957)
Drug	3 (3.1)	9 (1.9)	0.430	1.770 (0.462–6.774)
Surgical procedures				
Tonsillectomy	72 (73.5)	33 (6.8)	< 0.001	38.679 (21.664–69.058)
Adenoidectomy	52 (53.1)	48 (9.9)	< 0.001	10.443 (6.326–17.240)
Appendectomy	3 (3.1)	5 (1.0)	0.114	2.911 (0.675–12.560)
Tympanostomy	24 (24.5)	75 (15.4)	0.029	1.903 (1.117–3.240)
Other operative treatment	12 (12.2)	54 (11.1)	0.741	1.105 (0.564–2.163)
Other				
Regular medication	19 (19.4)	89 (18.3)	0.670	1.131 (0.745–1.716)

^*^OR not calculable due to zero events in one group

^a^Includes heterogeneous physician‑diagnosed conditions not captured in predefined categories

### Childhood infections

A significantly higher proportion of PFAPA patients reported a history of acute otitis media (AOM) in childhood compared with controls (36% vs 21%; OR 2.3, 95% CI 1.4–3.8). PFAPA patients also reported more often history of bronchitis than controls (4% vs 1%; OR 4.0, 95% CI 1.1–14.9). Hospitalization due to infections was more common among PFAPA patients than controls (30.2% vs 17.9%; OR 1.9, 95% CI 1.2–3.2). Fungal infections were also reported more frequently by PFAPA patients than controls (8.5% vs 3.7%; OR 2.4, 95% CI 1.0–5.9), (Table 2).

### Surgical procedures

Tonsillectomy was more common among PFAPA patients than controls (73.5% vs 6.8%; OR 38.7, 95% CI 21.7–69.1). Two cases underwent tonsillectomy for reasons unrelated to PFAPA. Because adenoidectomy is frequently performed in conjunction with tonsillectomy, its prevalence was also higher among PFAPA patients than controls (53.1% vs 9.9%; OR 10.4, 95% CI 6.3–17.2). PFAPA patients additionally reported having undergone tympanostomy more often than controls (24.5% vs 15.4%; OR 1.9, 95% CI 1.1–3.2). There were no differences between the groups in other operations (Table 3).

**Table 3:** Comparison of lifetime infection history in 98 PFAPA patients and 487 controls. Odds ratios are adjusted for age at data collection, sex, and social class of the family based on the father’s education (aOR). The number of respondents varies among cases (*n* = 93–98) and controls (*n* = 473–487)

	PFAPA group *N* (%), *n* = 98	Controls *N* (%), *n* = 487	*P* value	aOR (95% CI)
Infections				
Recurrent otitis media	34 (36.2)	101 (20.9)	0.001	2.317 (1.428–3.759)
Recurrent bronchitis	4 (4.2)	6 (1.2)	0.042	4.032 (1.088–14.940)
Pneumonia during lifetime	13 (14.0)	41 (8.7)	0.111	1.789 (0.907–3.531)
Other infection that was treated at the hospital	29 (30.2)	85 (17.9)	0.006	1.925 (1.169–3.168)
Herpes simplex	1 (1.1)	15 (3.1)	0.260	0.317 (0.041–2.442)
Fungal infections	8 (8.5)	18 (3.7)	0.041	2.437 (1.010–5.877)
Oral candidiasis	1 (1.1)	2 (0.4)	0.424	2.820 (0.246–32.316)
Diaper/intimate area	4 (4.3)	8 (1.7)	0.107	2.595 (0.743–9.066)
Between toes	2 (2.1)	7 (1.5)	0.629	1.382 (0.279–6.842)
Other topical	1 (1.1)	4 (0.8)	0.823	1.454 (0.158–13.372)

### Risk factors

Regarding the environmental factors studied, breastfeeding rates or the duration of breastfeeding did not differ significantly between PFAPA patients and controls. We found no differences between the groups regarding special diet, use of vitamin D or herbal products, nor having a pet (Table 4).

**Table 4:** Risk factors at the time of the questionnaire of 98 PFAPA patients and 487 controls. Odds ratios are adjusted for age at time of data collection, sex, and the social class of the family based on the father’s education

Environmental factor	PFAPA group *N* (%), *n* = 98	Controls *N* (%), *n* = 487	*P* value	aOR (95% CI)
Having siblings	90 (91.8)	452 (92.8)	0.735	0.890 (0.398–1.988)
Being breastfed	96 (98.0)	459 (95.2, *n* = 482)	0.225	2.682 (0.617–11.655)
Mother smoking	19 (19.4)	87 (17.9)	0.721	0.977 (0.556–1.716)
Father smoking	31 (31.6)	152 (31.2)	0.935	0.874 (0.537–1.423)
Sibling smoking	3 (3.1)	36 (7.4)	0.117	0.337 (0.099–1.141)
Subject smoking	3 (3.1)	14 (2.9)	0.920	0.954 (0.262–3.467)
Use of vitamin D	89 (90.8)	418 (88.4, *n* = 473)	0.485	1.384 (0.656–2.923)
Use of herbal and naturopathic products	6 (6.3, *n* = 96)	24 (5.3, *n* = 453)	0.709	1.158 (0.442–3.034)
Attending daycare^a^	86 (87.8)	439 (90.1)	0.477	0.870 (0.439–1.721)
Ever having animals^b^	56 (57.1)	290 (59.5)	0.659	0.794 (0.502–1.256)
Following a special diet	18 (18.4)	74 (15.2)	0.431	1.263 (0.713–2.238)

^a^We considered attendance at daycare center or family daycare for children over and under the age of two

^b^The animals examined are as follows: cat, dog, rodent, aquarium, terrarium, cow, sheep, horse, and pig

## Discussion

In this controlled long-term follow-up study, we found that individuals with a history of childhood PFAPA did not appear to have an increased risk for most chronic autoinflammatory or autoimmune illnesses, apart from a higher prevalence of atopy. Furthermore, while patients with PFAPA reported a higher lifetime occurrence of common childhood infections, specifically recurrent AOM, bronchitis, and topical fungal infections, the overall long-term prognosis remained favorable. These findings suggest that susceptibility to PFAPA may reflect a transient vulnerability during early childhood rather than a persistent predisposition to a chronic disease.

Previous long‑term follow‑up studies have consistently demonstrated a favorable prognosis for PFAPA, with spontaneous resolution of symptoms in most patients and no evidence of long‑term sequelae [14, 18]. More recent cohort study with extended follow‑up confirms that while most patients achieve remission, a minority may experience persistent or attenuated symptoms into adolescence or adulthood, with decreasing disease activity over time [19]. In this study, we found no differences between the groups regarding autoimmune or other chronic diseases, ongoing medications, or prior surgeries apart from tympanostomy. These findings support our earlier observation that PFAPA is not associated with an increased burden of long-term illnesses [14]. Previously proposed environmental risk factors, such as maternal smoking, lower breastfeeding rates, and the presence of an aquarium at home, were not reinforced in the present study [16]. Exploratory analyses comparing tonsillectomized patients with those experiencing spontaneous resolution did not reveal differences in overall morbidity or identified risk factors; however, atopy, food allergies, and history of tympanostomy were more frequently reported in the tonsillectomy group. These findings should be interpreted cautiously and do not support a causal relationship.

There has been conflicting evidence regarding how frequently children with PFAPA experience other childhood infections compared with their peers. In one of the first large population-based studies, parents reported that PFAPA children had less common infections than their siblings; furthermore, no patients were diagnosed with arthritis, pleuritis, myositis, or meningitis [4]. Another study of 105 PFAPA patients stated that 46% of parents perceived their children as less susceptible to circulating viral respiratory and gastrointestinal infections, although specific diagnoses were not detailed [13]. A third study reported that 53% of parents believed their PFAPA children had fewer infectious illnesses (excluding PFAPA episodes) compared with other children, but again, no distinction of infections was made [12].

In previous studies regarding infections, recurrent otitis media and tympanostomy tube insertion were as common in children with PFAPA as in controls, and the overall incidence of recurrent ear infections was low [12, 18]. In our earlier long-term follow-up study, an average 9 years after PFAPA diagnosis, we reported that 119 PFAPA patients had experienced respiratory tract infections, AOM, and oral thrush more frequently than their peers. The use of antibiotics was also common in this cohort, consistent with findings from earlier studies [2, 14]. The present study again demonstrates that PFAPA children have a higher lifetime prevalence of AOM and consequently tympanostomies than controls. In the present study, PFAPA patients reported a significantly higher number of AOM episodes compared to controls. In the early stages of the disease, before PFAPA is diagnosed, fever episodes are often misattributed to common infections. Diagnosing AOM in young children is challenging, and the combination of high fever, irritability, and a reddened eardrum may lead to overdiagnosis. Tonsillectomy is effective in stopping fever episodes and an established treatment method in PFAPA explaining over-representation among cases in this study. Adenoidectomy is frequently performed at the same time accounting for its increased prevalence. The observed increase in physician‑diagnosed infections and infection‑related hospitalizations among PFAPA patients should be interpreted with caution. These findings may reflect increased healthcare use rather than true susceptibility to infections. Children with PFAPA and their families have frequent contact with healthcare services, which likely lowers the threshold for evaluation, diagnosis, and hospitalization. This increased medical surveillance may contribute to higher reported infection rates compared with controls. Consequently, the results may partly represent detection bias rather than underlying immunological vulnerability. This raises the question of whether patients with PFAPA are truly comparable to their peers in terms of infectious morbidity.

Fungal infections, particularly oral candidiasis, were more common in PFAPA patients than in controls in both the present study and our previous work [14]. This increased susceptibility to fungal infections may be associated with the higher prevalence of atopy among PFAPA patients or reflect higher antibiotic exposure related to recurrent febrile episodes prior to diagnosis, rather than intrinsic susceptibility. Although most patients reported corticosteroid use for PFAPA episodes, treatment details were limited, and corticosteroids were typically used intermittently. Therefore, their potential contribution to fungal infections or other long‑term outcomes cannot be determined. The prevalence of atopy in PFAPA patients has also been noted in earlier studies [20]. In this study, atopy was based on self‑reported information and may not fully reflect clinically verified allergic disease, leading to potential misclassification. Recent studies suggest that PFAPA pathogenesis involves inflammasome-mediated immune activation followed by T-cell-driven adaptive responses [9, 21, 22]. *Candida albicans* has been identified as a potent activator of the function of inflammasomes [23]. Furthermore, earlier studies indicated that *C. albicans* was significantly more prevalent in the tonsils of PFAPA patients compared to controls, suggesting it may serve as a potential trigger for PFAPA flare-ups [24].

The primary strengths of our study include a large clinical cohort of verified PFAPA cases and enough population-derived, matched controls for robust comparisons. The long-term follow-up enabled reliable reporting of later comorbidities. The PFAPA cohort represented the full clinical spectrum of the syndrome at the time of diagnosis, including patients who were treated with tonsillectomy as well as those whose symptoms resolved spontaneously. The inclusion of patients without classic local symptoms, aphthous stomatitis, cervical adenitis, or pharyngitis supports the generalizability of our findings to the broader PFAPA population.

Several limitations should also be acknowledged. Newer classification criteria, such as the Eurofever/PRINTO criteria, could not be systematically applied retrospectively. However, the inclusion criteria ensured a clinically homogeneous cohort consistent with PFAPA as diagnosed in routine practice. Moderate response rate in the PFAPA group (40%) and the low response rate in the control group (20%) may introduce non‑response bias. Families with persistent symptoms, comorbidities, or increased health concerns may have been more likely to participate, potentially leading to an overestimation of comorbidity prevalence and symptom burden. Conversely, individuals whose symptoms have resolved completely may be underrepresented among responders. Although basic demographic characteristics of responders did not differ markedly from non‑responders, we cannot exclude differences in unmeasured clinical factors. A minority of patients reported ongoing febrile episodes at follow‑up. Persistent disease activity may influence healthcare utilization and increase the likelihood of reporting comorbid conditions. Patients with active or recurrent symptoms may have more frequent healthcare contacts and heightened awareness of medical issues, potentially contributing to higher observed comorbidity rates. This possible confounding effect should be considered when interpreting the results. A further limitation of this study is that outcome data were self‑reported retrospectively. This introduces a potential risk of recall bias, particularly given the long follow‑up period as some participants with a history of PFAPA completed the questionnaire 10–15 years after their diagnosis. Although major diagnoses and healthcare contacts are generally well remembered, the accuracy of recalled symptom severity, timing of resolution, and minor comorbidities may be limited. No systematic validation against medical records or registers was performed. This may have resulted in misclassification or overestimation of persistent symptoms and prevalence of comorbidity. We suspect that overdiagnosis may partly explain the prevalence of ear infections in PFAPA patients. Research findings, at least partly, indicated co-occurrence of infections and operations within the same individuals. Although controls were originally matched to PFAPA patients, the matched design could not be fully incorporated into the analyses due to differential response rates, and groups were therefore analyzed as independent samples with adjustment for matching variables. Additionally, multiple outcomes were assessed without formal correction for multiple testing, increasing the risk of type I error.

Based on this study, children with a history of PFAPA appear to be as healthy as their peers, apart from a higher rate of acute otitis media, bronchitis, and infection‑related hospitalizations. Increased rates of diagnosed infections and hospitalizations in PFAPA patients may be influenced by healthcare‑seeking behavior and diagnostic practices, rather than reflecting true infection susceptibility. The increased prevalence of atopy and fungal infections observed in PFAPA patients is noteworthy and merits further investigation.

## Data Availability

No datasets were generated or analysed during the current study.

## Abbreviations

AOM: Acute otitis media
aOR: Adjusted odds ratio
CI: Confidence interval
PFAPA: Periodic fever, aphthous stomatitis, pharyngitis, and adenitis syndrome
